# Ice-olating a health scoring system for wild polar bears

**DOI:** 10.1093/conphys/coaf047

**Published:** 2025-07-01

**Authors:** Kristina A Muise

**Affiliations:** Royal Veterinary College, University of London, Hawkshead Lane, North Mymms, Hertfordshire AL9 7TA, UK

How do you tackle the challenge of measuring the effects of chronic stress on wild polar bears (*Ursus maritimus*)? Sarah Teman and colleagues (Teman *et al.,* 2025) aimed to answer that question by creating an ‘allostatic load index’ for polar bears. The allostatic load index is a health scoring system that catalogues the number of biomarkers of physiological processes that may be abnormal. A higher allostatic load indicates that the animal may have been exposed to chronic stress, such as changes in their natural environment or changes in social dynamics with other animals. Generally, animals with a higher allostatic load index have an increased risk for diseases and death.

For their study, the team investigated the southern Beaufort Sea sub-population of polar bears (Fig. 1) located in northern Alaska, USA, and northwest Canada, which face many chronic stressors brought on by climate change and human actions. Hotter temperatures from a changing climate are leading to substantial decreases in sea ice cover, which is the bears’ natural hunting ground. As a result, polar bears move to land to find food, encountering more diseases and humans. Additionally, the bears are exposed to various heavy metals and chemicals in their habitat that can have negative effects on their health.

**Figure 1:**
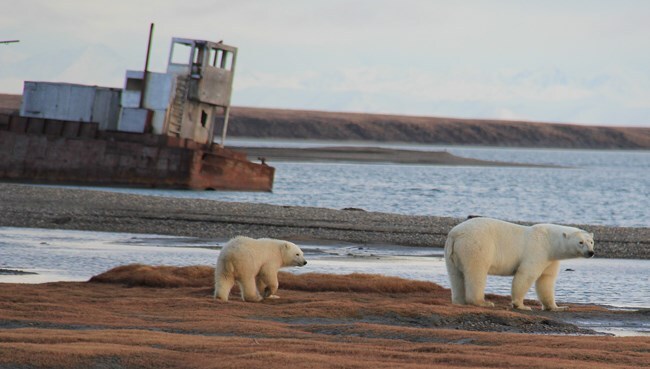
Adult female polar bear and cub in Kaktovik, Alaska. Image credit: USGS

Every year during a 35-year period (1983–2018), researchers from the U.S. Geological Survey captured polar bears and collected blood and hair samples. The bears’ body mass, body length, age and sex were also recorded. The bears were marked and then released back to the wild. Afterwards, Teman and colleagues used the blood and hair samples collected during the long-term study to measure different metabolic, neuroendocrine and immunological biomarkers of physiological processes in the bears. The team then compiled the allostatic load index for each bear, and linked it to the bear’s body condition, age, habitat and role in the family structure (such as mother bears, cubs or males).

The team found that the allostatic load index did not vary much across many scenarios, such as for bears with different body masses, or for the entire population throughout the 35-year study period. However, in some scenarios, the allostatic load index did vary. For example, in female bears that did not have cubs, the younger females had higher allostatic load indexes compared to older female bears—possibly reflecting stress from being inexperienced. Also, adult female bears without cubs that used land in the previous summer prior had a higher allostatic load index than those that used sea ice. The difference in allostatic load index suggests that land use is associated with higher stress in female bears, which could lead to an increased risk of disease and death.

Overall, Teaman and colleagues successfully created an allostatic load index to use in wild polar bears. The health information could be used to better assess how the bears are responding to potentially harmful events in their habitat that may threaten their survival or reproduction, which then can be included into future conservation management and policies.

## Notes

**Editor:** Ashleigh Donaldson
